# Evolution of Fagan's Nomogram; a Commentary

**Published:** 2016

**Authors:** Abdelrahman Ibrahim Abushouk

## Introduction

Dear Editor

I read with interest your paper entitled "Pre and post-test probabilities and Fagan’s nomogram" (1). I would like to add a note concerning an update on Fagan’s Nomogram. Generally, the basic idea of most nomograms is having the scales of 3 variables in a manner that if you draw a straight line between 2 values, the 3^rd^ value is found where the line intersects the 3^rd^ scale (2). They were initially developed in the 1980s by Maurice D'Ocange. Nomograms remained popular in medical practice until the invention of pocket calculators and computers. Their use increased again with the introduction of evidence-based medicine in clinical practice.

In a letter to the New England Journal of Medicine in July 1975, Dr Terry Fagan displayed a test characterization tool that went on to carry his name as the Fagan's nomogram (3). This nomogram is a simple application of the Bayes’ theorem, which establishes a rule to calculate the post-test probability of a disease.

However, Fagan's nomogram had a set of drawbacks that limited its use in clinical practice. These drawbacks included:

1- The original Bayes’ theorem is designed to deal with odds ratios, not probabilities, so algebraic conversion is needed to calculate probability. 

2- Most diagnostic tests are characterized in terms of sensitivity & specificity in the literature, which need special equations to be converted into likelihood ratios (4).

**Figure 1:**
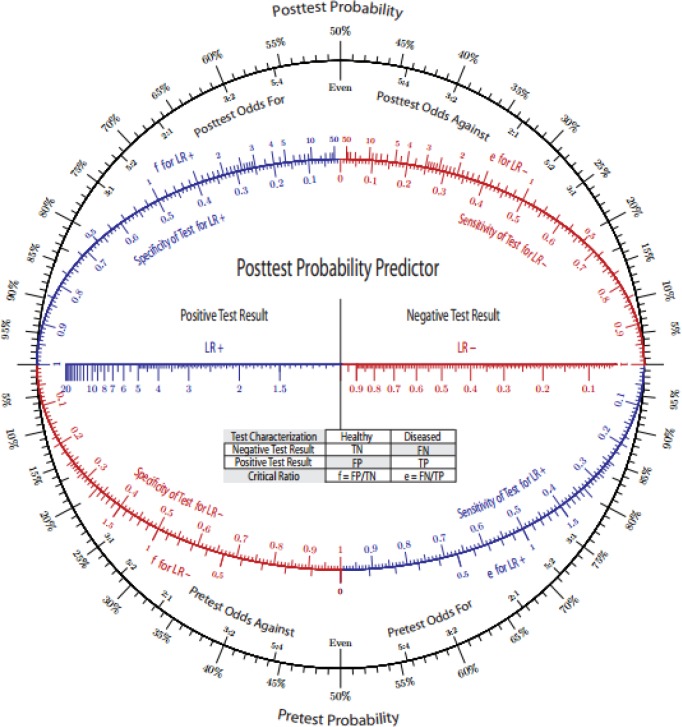
The modern Bayes’ theorem nomogram with an example of probability calculation as shown by the green line

Noticing these difficulties, in 2011, a group of researchers published a modern version of the nomogram that they named "Bayes’ theorem nomogram" (figure 1). The new nomogram targeted the former problems using:

A- Parallel lines for probability and odds on each side of the nomogram figure.

B- The inner lip along the entire circle contains values for sensitivity and specificity that can be connected to calculate the likelihood ratio of a certain diagnostic test (5).

As illustrated in figure (A), a pretest probability of 18% and a likelihood ratio (LR+) of 2.8 for a diagnostic test would give a posttest probability of 38%. 

Further advantages of the modern nomogram include:

- In Rare disorders, having a very low pretest probability implies performing a diagnostic test with a fairly high LR (+). In Fagan’s nomogram, the high values of LR are compressed in a tight portion over that scale (4), while in this model; a more spaced representation of high LR is feasible. 

- Replacing the linear form with a circular one works better for complex diagnostic protocols where addition of multiple arrows for different diagnostic tests may be required (5).

Considering the mentioned superiorities of the Bayes’ theorem nomogram over the conventional Fagan's nomogram, it is highly recommended for clinicians to use it in conducting diagnostic protocols and formulating therapeutic plans.
